# Unusual severe hypoxemia due to unilateral pulmonary edema after conventional cardiopulmonary bypass salvaged by veno-venous extracorporeal membrane oxygenation: a case report

**DOI:** 10.1186/s40981-023-00656-2

**Published:** 2023-10-07

**Authors:** Masataka Fukuda, Hiroaki Sakai, Keito Koh, Sonoko Sakuraba, Nozomi Ando, Masakazu Hayashida, Izumi Kawagoe

**Affiliations:** 1 Department of Anesthesiology and Pain Medicine, Juntendo University Hospital, Tokyo, 113-8431 Japan; 2 Department of Anesthesia, Fujieda Municipal General Hospital, Shizuoka, Japan; 3 Department of Anesthesiology and Pain Medicine, Juntendo University Shizuoka Hospital, Shizuoka, Japan

**Keywords:** Cardiopulmonary bypass, Re-expansion pulmonary edema, Unilateral pulmonary edema, Veno-venous extracorporeal membrane oxygenation

## Abstract

**Background:**

We report a case in which veno-venous extracorporeal membrane oxygenation (V-V ECMO) saved the life of a patient who developed severe hypoxemia due to unusual unilateral pulmonary edema (UPE) after cardiopulmonary bypass (CPB).

**Case presentation:**

A 69-year-old man underwent aortic valve replacement and coronary artery bypass grafting. Following uneventful weaning off CPB, he developed severe hypoxemia. The ratio of arterial oxygen tension to inspired oxygen fraction (PaO_2_/FiO_2_) decreased from 301 mmHg 5 min after CPB to 42 mmHg 90 min after CPB. A chest X-ray revealed right-sided UPE. Immediately established V-V ECMO increased PaO_2_/FiO_2_ to 170 mmHg. Re-expansion pulmonary edema (REPE) was likely, as the right lung remained collapsed during CPB following the accidental opening of the right chest cavity during graft harvesting.

**Conclusions:**

V-V ECMO was effective in improving oxygenation and saving the life of a patient who had fallen into unilateral REPE unusually developing after conventional CPB.

## Background

Acute pulmonary edema after cardiac surgery develops due to cardiogenic and non-cardiogenic causes and occasionally leads to severe hypoxemia that cannot be managed with mechanical ventilation alone [1, 2]. Unilateral pulmonary edema (UPE) after on-pump minimally invasive cardiac surgeries (MICS) has been increasingly reported [3]. However, reports on UPE after conventional on-pump cardiac surgeries remain very rare [4]. Herein, we report a case in which veno-venous extracorporeal membrane oxygenation (V-V ECMO) saved the life of a patient with severe hypoxemia due to UPE that developed acutely after weaning off conventional cardiopulmonary bypass (CPB).

## Case presentation

A 69-year-old man (162 cm, 48.5 kg), with a history of coronary artery disease (CAD) previously treated with percutaneous transluminal coronary angioplasty, hypertension as well as diabetes mellitus under medication, and end-stage diabetic nephropathy on hemodialysis, was scheduled for aortic valve replacement (AVR) and coronary artery bypass grafting (CABG) for newly diagnosed severe aortic stenosis (AS) and recurrent CAD. Preoperative transthoracic echocardiography showed a global left ventricular ejection fraction (in the presence of regional hypokinesis) of 53%, left ventricular diastolic/systolic dimensions of 37/27 mm, and AS due to calcified valve leaflets, with an aortic valve area (AVA) of 0.5 cm^2^ and indexed AVA of 0.19 cm^2^/m^2^. Coronary angiography showed 90% stenotic lesions in the right coronary artery (RCA) and left circumflex artery (LCX).

After the establishment of an arterial line, general anesthesia was induced with midazolam 4 mg and fentanyl 400 µg. Vecuronium bromide 8 mg was given to facilitate tracheal intubation. A central venous catheter and pulmonary artery catheter (PAC) were placed via the right internal jugular vein. Transesophageal echocardiography (TEE) was also used. General anesthesia was maintained with propofol 2 mg/kg/h and remifentanil 0.3 µg/kg/min. Following a median sternotomy, the right internal thoracic artery (RITA) graft and saphenous vein graft (SVG) were harvested. During RITA harvesting, the right chest cavity was accidentally opened but was subsequently left opened until immediately before chest closure. After graft harvesting, CPB was started with the arterial cannula placed in the ascending aorta and venous cannulas placed in the superior and inferior vena cava (IVC). First, on-pump beating CABG was performed, including a RITA-LCX anastomosis and two sequential SVG-RCA anastomoses. Then, the ascending aorta was cross-clamped, and AVR was performed with a Trifecta biological valve. During CPB, the patient received transfusions of 12 units of red cell concentrates and 4 units of irradiated fresh-frozen plasma (FFP).

After completion of the surgical repair, adequate de-airing of the heart was facilitated with a manual lung inflation maneuver and volume loading of the heart. Prior to weaning from CPB, mechanical ventilation was resumed with an inspired oxygen fraction (FiO_2_) of 1. The patient was successfully weaned off CPB with some inotropic support. His pulmonary artery pressure (PAP) remained normal, and cardiac index (CI) remained at approximately 2.5 L/min/m^2^. TEE revealed the well-functioning artificial valve, well-preserved left ventricular wall motion, trivial mitral regurgitation (MR), and no enlargement of the left atrium (LA). A significant volume of retained blood was evacuated from the right thoracic cavity. The patient was uneventfully weaned off CPB. The durations of CPB and aortic cross-clamping were 251 min and 127 min, respectively.

**Fig. 1:**
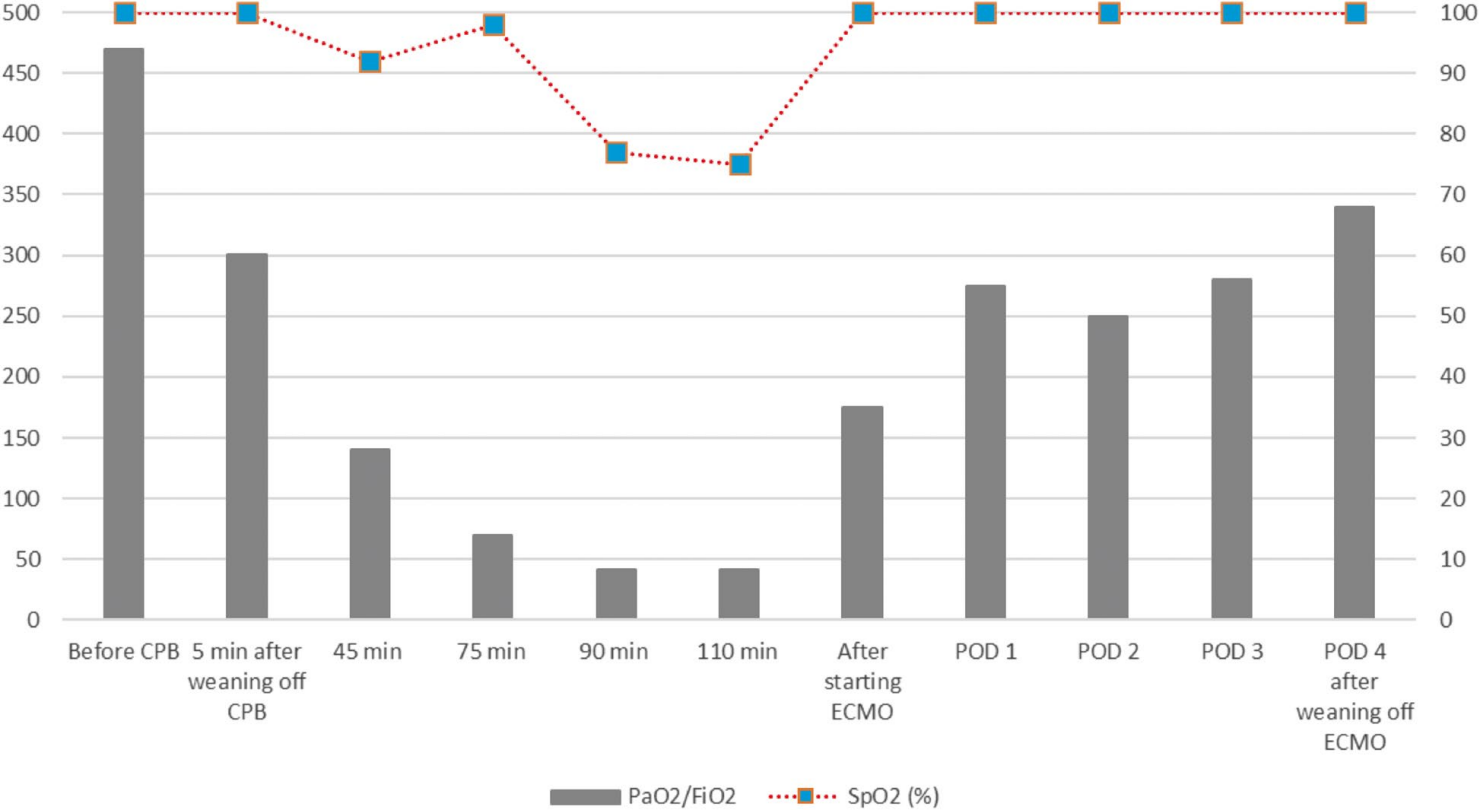
PaO_2_/FiO_2_ ratio and SpO_2_ during and after surgery. PaO_2_/FiO_2_, ratio of arterial oxygen tension to inspired oxygen fraction; SpO_2_, percutaneous arterial oxygen saturation; CPB, cardiopulmonary bypass; ECMO, extracorporeal membrane oxygenation; POD, postoperative day

**Fig. 2:**
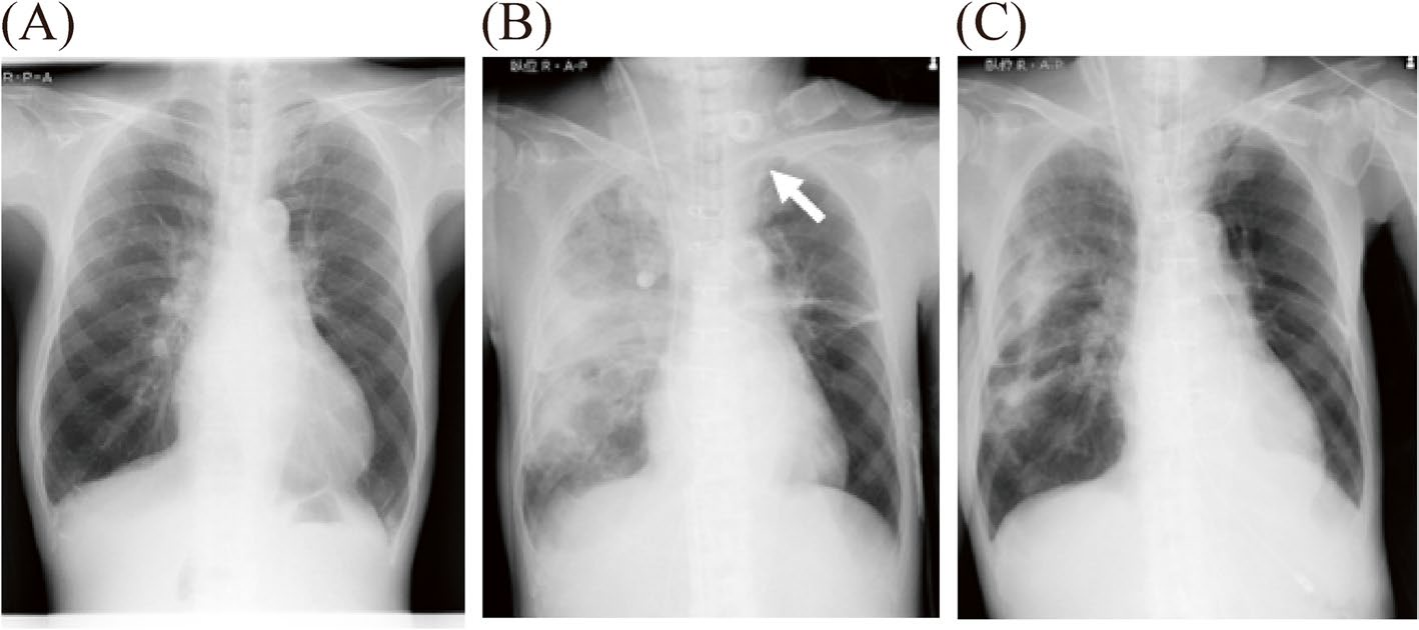
Chest X-rays **A** before surgery, **B** after chest closure, and **C** on postoperative day 5. The chest X-ray taken immediately after chest closure (**B**) showed diffuse unilateral pulmonary consolidation in the right lung, with no increase in cardiothoracic ratio, consistent with non-cardiogenic, unilateral pulmonary edema. That taken on POD 5 (**C**) showed improvement in unilateral pulmonary consolidation. The white arrow depicted in **B** indicates a return cannula placed via the left internal jugular vein

**Fig. 3:**
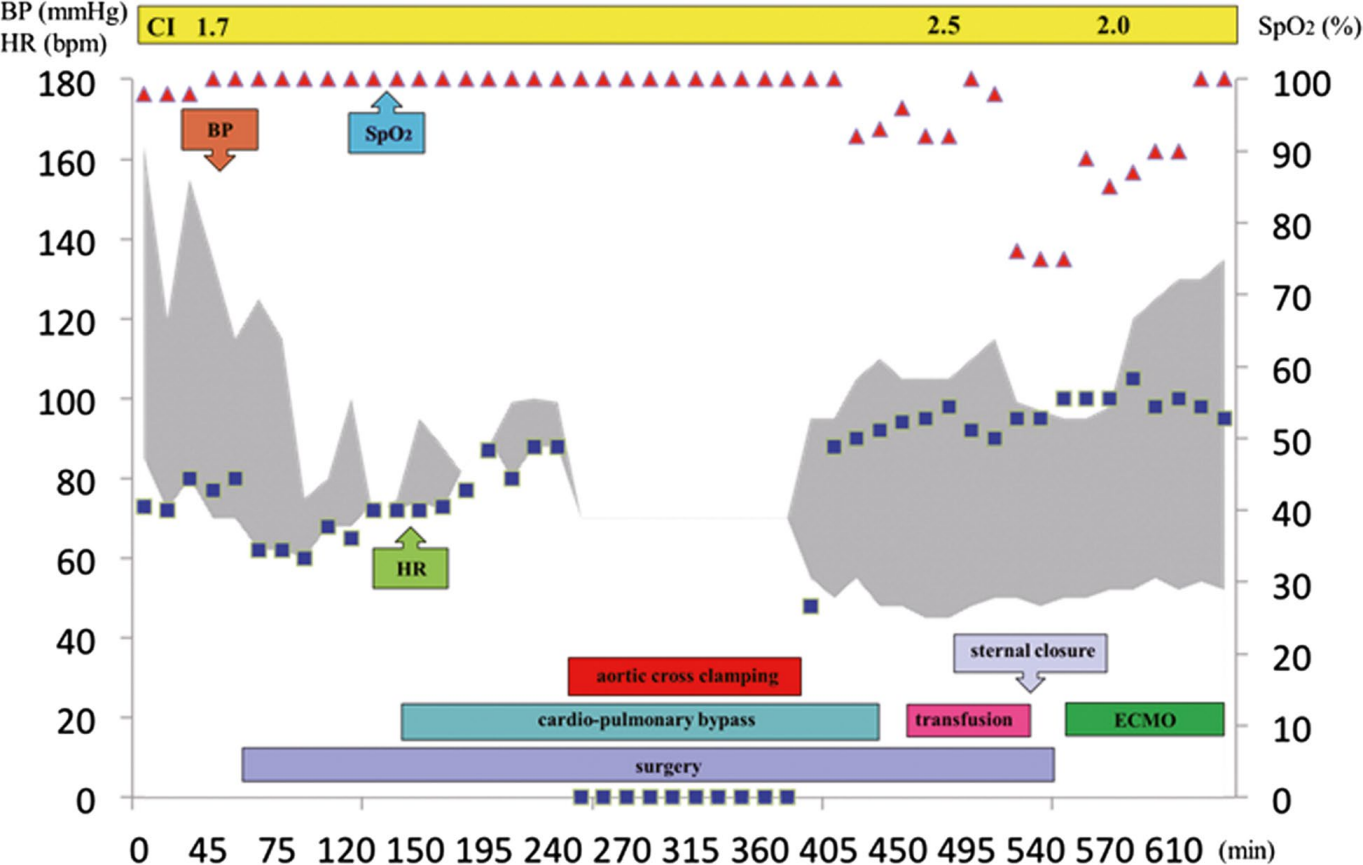
Hemodynamic changes before, during, and after surgery. Anesthesia starts from 0 on the horizontal axis. BP, blood pressure; HR, heart rate; CI, cardiac index; SpO , percutaneous arterial oxygen saturation; ECMO, extracorporeal membrane oxygenation

After administration of protamine, 4 units of irradiated FFP and 20 units of platelet concentrates were transfused. Shortly after the start of the transfusion, however, he developed progressive hypoxemia. The ratio of arterial oxygen tension to FiO_2_ (PaO_2_/FiO_2_) decreased from 301 mmHg 5 min after CPB to 140 mmHg 45 min after CPB and, further, to 42 mmHg 90 min after CPB (Fig. 1) . As soon as the surgical wound was closed 475 min after the start of surgery, anesthesiologists inserted a return cannula into the right atrium via the left internal jugular vein, while surgeons inserted a drainage cannula into the IVC via the right femoral vein to establish V-V ECMO. A chest X-ray showed diffuse unilateral pulmonary consolidation in the right lung with no increase in a cardiothoracic ratio, indicative of non-cardiogenic UPE (Fig. 2). V-V ECMO with a flow rate of 2 L/min successfully increased PaO_2_/FiO_2_ to 170 mmHg (Figs. 2 and 3).

The patient was transferred to the intensive care unit. Continuous hemodiafiltration was started via a catheter placed in the left femoral vein. The improvement in oxygenation was maintained with V-V ECMO and pressure-controlled ventilation employing a lung-protective strategy (Fig. 1). The patient was weaned off V-V ECMO on postoperative day (POD) 3 (Fig. 1) and was extubated on POD 7. The patient was, however, re-intubated due to aspiration on POD 7 and re-extubated on POD 13. He returned to the general ward on POD 17 and was subsequently discharged in an ambulatory condition on POD 78. Neither antihuman leukocyte antigen (HLA) antibody nor anti-granulocyte antibody was detected in samples of the transfused blood products.

## Discussion

Acute cardiogenic or non-cardiogenic pulmonary edema sometimes develops in patients undergoing major cardiovascular surgery, although most cases present with bilateral pulmonary edema (BPE) [5]. Reportedly, UPE, which accounts for 2.1% of cardiogenic pulmonary edema (CPE) cases, usually occurs in the right lung [6]. Although there is no specifically established treatment for UPE that differs from BPE, UPE is associated with more severe conditions requiring mechanical ventilation and more intensive inotropic support [6]. The major cause of cardiogenic UPE is MR due to its propensity to flow anatomically into the right superior pulmonary vein [7]. Another underlying mechanism of right-sided UPE is a difference in interstitial flow in the lungs due to the right-sided lymphatic vessels having a smaller aperture and a longer distance to the thoracic duct than left-sided lymphatic vessels [8, 9].

In our patient, UPE occurred in the right lung. However, CPE was unlikely, since TEE did not reveal significant MR or LA enlargement, and instead revealed well-preserved left ventricular wall motion, while the PAC revealed normal PAP and CI, and chest X-ray revealed neither the increased cardiothoracic ratio nor “butterfly pattern” typically seen in CPE.

UPE can develop also due to other pathological conditions, such as acute pulmonary emboli, re-expansion pulmonary edema (REPE), and external compression of the pulmonary vasculatures by a tumor or hematoma, resulting in asymmetrical inflow to the pulmonary arteries or outflow to the pulmonary veins [10]. In our case, the right lung was left collapsed during CPB after discontinuation of mechanical ventilation, since the right chest cavity was opened during RITA graft harvesting. Furthermore, blood retention in the cavity might have aggravated the collapse. Therefore, REPE due to rapid re-expansion of the right lung following its collapse likely caused right-sided UPE. Typically, REPE develops only after the lung collapsed for 72 h or longer is rapidly reinflated [11]. However, since on-pump MICS via right mini-thoracotomies using one-lung ventilation are increasingly being performed, it has become evident that right-sided unilateral REPE can develop much earlier after CPB [12, 13]. Lung collapse is associated with the sequestration of leucocytes, and inflammatory activity is triggered when oxygen is supplied during re-expansion and concomitant reperfusion of the lung [14, 15]. This inflammatory response might be enhanced by multiple factors during cardiac surgery [12]. The inflammatory response to re-expansion and reperfusion can be aggravated by the generalized inflammatory response generated by CPB [12], as suggested by the association between prolonged CPB time and the development of UPE in MICS [13, 14]. The FiO_2_ set at 1.0 upon resuming ventilation might have worsened the inflammatory reaction during re-expansion, though we reduced the FiO_2_ to 0.5 immediately after measuring blood gas 5 min after CPB. Furthermore, the restricted bronchial artery blood flow during CPB might worsen lung ischemia [16]. Therefore, the combination of right lung collapse and prolonged CPB time might have resulted in the unilateral REPE in our case, although unilateral REPE has rarely been reported in patients undergoing conventional on-pump cardiac surgery [4], unlike those undergoing on-pump MICS [12, 13].

Since UPE occurred after the start of blood transfusion in our patient, we also considered the possibility of transfusion-related acute lung injury (TRALI) [17–19]. However, anti-HLA antibody or anti-granulocyte antibody, which reportedly triggers lung microvascular endothelial damage [19], was not detected in samples of transfused blood products. Additionally, TRALI usually presents with BPE [18]. Therefore, TRALI less likely caused UPE than REPE in our case.

The use of ECMO is indicated when a poor therapeutic response to conventional mechanical ventilation and a high mortality rate are predicted in patients with reversible lung injury [20, 21]. When considering the indication for ECMO, it is recommended to estimate the Murray score, in which a score of 2.5 or more indicates “severe” lung injury [21, 22]. Our patient was indicated for ECMO, since he was highly likely to die from severe hypoxemia without using ECMO, given his Murray score estimated to be 2.75 and PaO_2_/FiO_2_ as low as 42 mmHg.

In conclusion, we reported a rare case of UPE with severe hypoxemia that developed after conventional on-pump cardiac surgery. REPE seemed the likely cause of the UPE. V-V ECMO was quite effective in improving oxygenation and saving the patient’s life.

## Data Availability

The datasets related to this report are available from the corresponding author upon reasonable request.

## Abbreviations

AS: Aortic stenosis
AVA: Aortic valve area
AVR: Aortic valve replacement
BPE: Bilateral pulmonary edema
CABG: Coronary artery bypass grafting
CAD: Coronary artery disease
CI: Cardiac index
CPB: Cardiopulmonary bypass
CPE: Cardiogenic pulmonary edema
FFP: Fresh-frozen plasma
FiO_2_: Inspired oxygen fraction
IVC: Inferior vena cava
LA: Left atrium
LCX: Left circumflex artery
MICS: Minimal invasive cardiac surgery
MR: Mitral regurgitation
PAC: Pulmonary artery catheter
PaO2/FiO2: Ratio of arterial oxygen tension to fraction of inspired oxygen
PAP: Pulmonary artery pressure
POD: Postoperative day
RCA: Right coronal artery
REPE: Re-expansion pulmonary edema
RITA: Right internal thoracic artery
SVG: Saphenous vein graft
TRALI: Transfusion-related acute lung injury
TEE: Transesophageal echocardiography
UPE: Unilateral pulmonary edema
V-V ECMO: Veno-venous extracorporeal membrane oxygenation
